# Formulation Development and Characterization of pH Responsive Polymeric Nano-Pharmaceuticals for Targeted Delivery of Anti-Cancer Drug (Methotrexate)

**DOI:** 10.3389/fphar.2022.911771

**Published:** 2022-06-30

**Authors:** Farhad Ullah, Zafar Iqbal, Amjad Khan, Saeed Ahmad Khan, Lateef Ahmad, Amal Alotaibi, Riaz Ullah, Muhammad Shafique

**Affiliations:** ^1^ Department of Pharmacy, University of Peshawar, Peshawar, Pakistan; ^2^ Department of Pharmacy, Kohat University of Science and Technology (KUST), Kohat, Pakistan; ^3^ Department of Pharmacy, University of Swabi, Swabi, Pakistan; ^4^ Department of Basic Science, College of Medicine, Princess Nourah Bint Abdulrahman University, Riyadh, Saudi Arabia; ^5^ Medicinal, Aromatic, and Poisonous Plants Research Center, Department of Pharmacognosy, College of Pharmacy, King Saud University, Riyadh, Saudi Arabia; ^6^ Department of Pharmaceutical Sciences, College of Pharmacy-Boys, Al-Dawadmi Campus, Shaqra University, Shaqra, Saudi Arabia

**Keywords:** Eudragit, methotrexate, targeted drug delivery, polymeric nanoparticles, solvent evaporation method

## Abstract

Oral administration of pH sensitive/stimuli responsive nanoparticles are gaining importance because of the limited side effects, minimum dose and controlled drug release. The objective of this study was to develop and evaluate pH sensitive polymeric nanoparticles for methotrexate with the aim to maximize the drug release at target site. In the presented study, pH sensitive polymeric nanoparticles of methotrexate were developed through modified solvent evaporation technique using polymer Eudragit S100. Different process parameters like drug to polymer ratio, speed of sonication, concentration of surfactant and time of sonication were optimized by evaluating their effects on particle size, PDI, zeta potential, entrapment/encapsulation efficiency. The developed formulations were evaluated for their size, polydispersity (PDI), zeta potential, encapsulation efficiency, XRD, scanning electron microscopy, *in-vitro* drug release and stability studies. Best results were obtained with poloxamer-407 and PVA and were selected as surfactants. Physicochemical characterization of the developed formulations showed that the particle size lies in the range 165.7 ± 1.85–330.4 ± 4.19, PDI 0.119 ± 0.02–0.235 ± 0.008, zeta potential −0.163 ± 0.11–−5.64 ± 0.36 mV, and encapsulation efficiency more than 61%. The results of scanning electron microscopy revealed that nanoparticles have regular geometry with spherical shape. Initially the drug release occur through diffusion followed by erosion. The present studies showed that MTX-ES100 nanoparticles prepared during this study have the desired physicochemical properties, surface morphology and release characteristics used to target the desired organs.

## 1 Introduction

Drug administration by oral route is the most ideal owing to its simplicity, convenience, minimal pain and suitability, especially for chronic therapy (Sarmad et al., 2021). It is expected to solve the noncompliance-related problems associated with injections and other aggressive dosage forms (Benish et al., 2021). In addition, oral formulations have unique advantages for both physicians and industry, such as flexible dosing schedules, less demands on staff, reduced costs through less hospital or clinic visits, and less expensive production costs (Sharma et al., 2016; Amjad, 2019). However, orally delivered drugs are exposed to extreme conditions and variable pH throughout the GIT which can adversely affect drug absorption. Some drugs like peptides and protein, may be degraded by digestive enzymes (Late et al., 2009) and by variation in pH of the gastrointestinal (GI) tract. In GIT the pH varies from highly acidic in the stomach (pH 1–3) to neutral or slightly alkaline in the duodenum (pH 6) and along the jejunum and ileum (pH 6–7.5) (Hadi et al., 2012; Amjad et al., 2016) and can result in hydrolysis, oxidation or de-amidation of protein. The intestinal epithelium is also a barrier to the absorption of hydrophilic macromolecules such as peptide, proteins, nucleic acids, and polysaccharides due to their hydrophilicity and high molecular weight, which makes it difficult to across the cell membranes (Hazal et al., 2021). Due to possibility of low bioavailability after oral administration of many drugs, such as proteins, it has become a challenge to achieve consistent and adequate bioavailability for their oral administration (Dilpreet, 2021). Of varied methods for overcoming the barriers, pH triggered release mechanisms are extensively used in oral administration. The pH-responsive carriers for oral drug delivery have been proven to enhance the stability of drug delivery in stomach and achieve controlled release in intestines. A pH-responsive and colon-specific capsule which is potential to be used as a reliable carrier for colon-specific drug delivery has been reported (Bohrey et al., 2016).

Polymeric nanoparticles (NPs) have been extensively studied for oral delivery as it can protect encapsulated drugs from the low pH environment, drug efflux pumps, and enzymatic degradation. Recently, through cellular targeting with surface-functionalized ligands, transepithelial transport, and greater gastric retention, pH-responsive mechanisms have been included in novel nanomedicines to improve systemic exposure. One widespread approach to realize organ-specific drug release is to prepare NPs that exhibit pH-responsive swelling. For instance, when using acrylic-based polymers (e.g., PMAA), NPs retain a hydrophobic, collapsed state in the stomach because of carboxyl protonation. After moving though gastric passage, increasing pH results in NPs swelling due to the ionization of carboxyl groups and hydrogen bond breakage (Madani et al., 2018).

Eudragits, is poly(methacrylic acid-co-methyl acrylate) copolymers, and is widely used in formulation of pH-responsive NPs. Depending upon their solubility, Eudragit are classified as;

• Eudragit E100: Eudragit E100 is a cationic copolymer which dissolves in stomach,
• Eudragit S100: Eudragit S100 is an anionic copolymers and dissolves at pH4.5
• Eudragit L100: Eudragit L100 is an anionic copolymers which dissolves at pH7

Due to variability in their solubility at different pH eudragit can be used in formulation of pH responsive drug delivery system for oral administration (Yoo et al., 2011). Objective of the study was to develop pH responsive, colon targeted drug delivery system for oral administration of anti-cancer drug (methotrexate). In the present study, Eudragit S100 based nanoparticles were prepared by solvent evaporation technique and evaluated for various quality control parameters and pH dependent drug release.

## 2 Materials and Methods

### 2.1 Materials and Solvents

Methotrexate (purity ≥99.9%) (Huzhou Zhanwang Pharma Co., Ltd., China), Eudragit^®^ S100 (Evonik, Germany), Poloxamer-407 (Sigma-Aldrich), Poloxamer-407 (POL) (Merck, Germany), Cetyl Trimethyl Ammonium Bromide (CTAB) (Merck, Germany), Poy vinyl alcohol (PVA)Sigma-Aldrich, Sodium Bicarbonate (Fluka) (purity 99.95%), Sodium dodecyl sulphate (SDS) (Sigma-Aldrich), Sodium Chloride (NaCl), Dialysis Tubing (Size 6 Inf, Dia “27/32,” 21.5 mm; 30 M) (Sigma-Aldrich) (MWCO: 12–14 kDa), Potassium Chloride (KCl) (Scharlau Chemie Spain), (Na_2_HPO_4_) Disodium Hydrogen Phosphate (Scharlau Chemie Spain), (KH_2_PO_4_) Potassium Di-hydrogen Phosphate (Sigma-Aldrich).

### 2.2 Preparation of Drug Loaded Polymeric Nanoparticles

**FIGURE 1:**
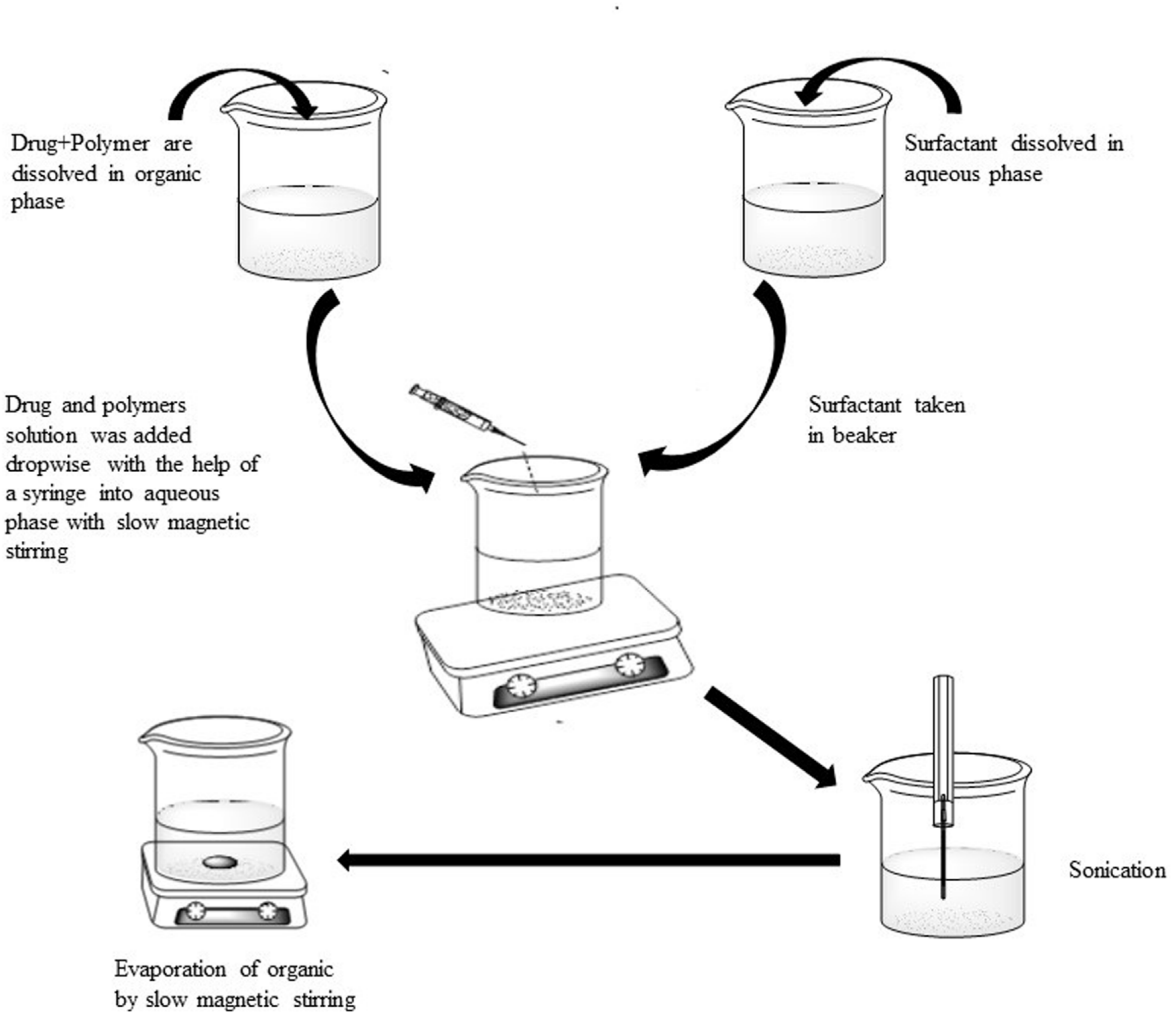
Schematic presentation of the process of preparation of methotrexate loaded polymeric nanoparticles.

Polymeric nanoparticles of methotrexate were formulated by modified emulsion solvent evaporation technique (Nasef et al., 2015). Different stabilizers like POL, cetyl trimethyl ammonium bromide, polyvinyl alcohol and sodium dodecyl sulphate (SDS) were used in different concentrations (0.5%, 0.25%, and 0.125%). The solutions of surfactant were formed by solubilizing in distilled water and used constant volume (10 ml) of solutions of different concentrations. Both the drug and polymer (Eudragit) were dissolved in methanol (5 ml) and an aliquot (5 ml) was added dropwise to aqueous solution of surfactant (10 ml) under continuous magnetic stirring. After complete addition of organic phase, the resulting mixture was subjected to sonication at 99% amplitude for 3 min with the help of probe sonicator (Soniprep, 150 instruments; Sanyo, United Kingdom) fitted with exponential microprobe, having an end diameter of 3 mm. The resultant emulsion was then stirred at low speed with magnetic stirrer in order to remove organic solvents completely and centrifuged at 15,000 rpm for 30 min at 4°C to collect the drug loaded nanoparticles. The obtained nanoparticles were washed three times with double distilled water and lyophilized. Figure 1 shows schematic presentation of the process of preparation of methotrexate loaded polymeric nanoparticles while detailed composition of different formulations is presented in Table 1.

**TABLE 1:** Composition of methotrexate nanoparticles prepared by using Eudragit S100.

Code	Drug (mg)	Eudragit S100 (mg)	Polaxamer 407 (%) (10ml)	PVA (%) (10ml)
MSX1	2	10	0.50	—
MSX2	2	10	0.25	—
MSX3	2	10	0.125	—
MSX4	2	20	0.50	—
MSX5	2	20	0.25	—
MSX6	2	20	0.125	—
MSX7	2	30	0.50	—
MSX8	2	30	0.25	—
MSX9	2	30	0.125	—
MSP1	2	10	—	0.50
MSP2	2	10	—	0.25
MSP3	2	10	—	0.125
MSP4	2	20	—	0.50
MSP5	2	20	—	0.25
MSP6	2	20	—	0.125
MSP7	2	30	—	0.50
MSP8	2	30	—	0.25
MSP9	2	30	—	0.125

All formulations are prepared at 25°C and 99% sonication speed.

### 2.3 Optimization of Process Variables

Different formulation and process variables like polymer concentration, surfactant concentration, type of surfactant, time of sonication, amplitude of sonication and drug concentration were then optimized for nanoparticles.

For optimization of polymer quantity, different drug to polymer ratios (10, 20, and 30 mg) were studied and their effect on results was evaluated. Different surfactant (POL, PVA, SDS, and CTAB) were tested for emulsification and the selected surfactant was studied at different concentrations (0.125%, 0.25%, and 0.5% w/v) to get the optimal results.

Emulsification was performed at different homogenization speeds (60%, 80%, and 99%) and effect on characteristics of the droplets were evaluated. Similarly, sonication time was varied between 1–6 min and its effect was evaluated.

To optimize the effect of amount of drug in the nanoparticles, variable amount of drug (2–6 mg) was added to the organic phase and its effect on encapsulation efficiency and other characteristics was evaluated. Details of optimization parameters are mentioned in Table 2.

**TABLE 2:** Effect of process variables on particle size and encapsulation efficiency of methotrexate nanoparticles with Eudragit S100.

Code	Drug (mg)	Eudragit S100 (mg)	Polaxamer 407 (%) (10 ml)	Temp (°C)	Sonication speed (%)	Sonication time (min)	CTAB (%) (10 ml)	SDS (%) (10 ml)	PVA (%) (10 ml)
FMVs1	2	20	0.25	25	60	3	—	—	—
FMVs2	2	20	0.25	25	80	3	—	—	—
FMVs3	2	20	0.25	25	99	3	—	—	—
FMVt4	2	20	0.25	25	99	1	—	—	—
FMVt5	2	20	0.25	25	99	3	—	—	—
FMVt6	2	20	0.25	25	99	6	—	—	—
FMVr7	2	20	—	25	99	3	—	0.25	—
FMVr8	2	20	—	25	99	3	0.25	—	—
FMVr9	2	20	—	25	99	3	—	—	0.25
FMVr10	2	20	0.25	25	99	3	—	—	—
FMVd11	2	30	0.25	25	99	3	—	—	—
FMVd12	4	30	0.25	25	99	3	—	—	—
FMVd13	6	30	0.25	25	99	3	—	—	—

### 2.4 Characterization of Polymeric Nanoparticles of Methotrexate

#### 2.4.1 Measurement of Particle Size and Polydispersibility Index

The nanoparticles size and PDI was evaluated through dynamic light scattering technique by means of Zeta Sizer (Zeta sizer Nano, ZS-90; Malvern Instruments Ltd., United Kingdom). The distilled water was added to the dispersion of nanoparticles when required. The nanoparticles were analyzed at scattering angle of 90° at room temperature (Nasef et al., 2015). The size of particles and PDI was determined through Malvern software. All the values were calculated three times and their mean and standard deviation were calculated.

#### 2.4.2 Measurement of Zeta Potential

Laser Doppler Micro-electrophoresis method was utilized for determining the zeta potential of nanoparticles utilizing Zeta Sizer Nano. Readings were taken three times and their mean and SD determined.

#### 2.4.3 Determination of Percent Encapsulation Efficiency

The percent drug entrapment efficiency of methotrexate (MTX) was calculated by centrifuging the nanoparticle suspension so as to separate nanoparticle from aqueous medium at 15,000 rpm for 30 min 25°C. The free drug present in supernatant collected after centrifugation was calculated at 295 nm using UV spectroscopy. The % EE was obtained as per Eq. 1.

$$\%EE=\frac{Mass\;of\;Drug\;\;in\;Nanoprticles}{Mass\;of\;Drug\;used\;in\;Formulation}\;\times 100\;$$ (1)

#### 2.4.4 Scanning Electron Microscopy

The surface morphology of prepared nanoparticles was determined through scanning electron microscopy, using Brass stub for sample preparation. The sample was placed on a double tape made of carbon that was attached to the stub. The excess quantity of sample was removed from the tape by blade. Then gold was coated on the surface of nanoparticles by a sputter coater (“Argon Sputtering,” “SPI Module” Control) for 90 s under vacuum produced by argon gas. The surface morphology of the sample was then confirmed by electron microscope (JSM-5910, Jeol Japan).

#### 2.4.5 X-Ray Diffraction

The X-ray diffraction patterns of different samples were measured by X-ray diffractometer so as to determine the nature of sample whether amorphous or crystalline. The x-ray diffraction pattern of the samples determined included; MTX, Poloxamer-407, PVA, Eudragit S100, and MTX-NPs. The instrument was run at 3° (2θ)–80° (2θ) angular range.

#### 2.4.6 *In Vitro* Drug Release

The *in-vitro* drug release profile of MTX from polymeric nanoparticles was determined using dialysis bag diffusion method. Dissolution media consisted of simulated gastric fluid (0.1 N HCl pH 1.2), simulated intestinal fluid (PB pH 6.5) and simulated colonic fluid (PB pH 7.4). Dissolution media (100 ml) was taken in a flask, de aerated and equilibrated to 37°C ± 2°C on a shaking water bath. Dialysis membrane containing nano suspension (1 ml) of MTX was dipped in dissolution media. The flask containing dissolution media was agitated at 60 ± 2 rpm. Samples (1 ml) were withdrawn at specific interval of time (0.25, 0.5, 1, 1.5, 2, 3, 4, 6, 8, 10, 12, 18, 24, 36, and 48) and quantity of drug release was determined through UV spectrophotometer at 295 nm. Volume of dissolution media was adjusted by same volume after each procedure of sampling held at same temperature. All samples were analyzed three time and their average and SD were calculated (*n* = 3). The dissolution medias were replaced depending on the time intervals assuming that the drug keep on passing the GIT. *In vitro* drug release was initiated in simulated gastric fluid (SGF) for first 2 h and then dissolution media was replaced with simulated intestinal fluid (SIF) for next 4 h. The dissolution medium was then interchanged with simulated colonic fluid (SCF) and drug release studies were continued for 42 h.

### 2.5 Freeze Drying and Selection of Cryoprotectant

The developed formulations were lyophilized using freeze drier (Telstar Cryodos 50, United States) to get dried methotrexate loaded nanoparticles for reconstitution and evaluation of stability. In order to optimize and select a suitable cryoprotectant, mannitol and sucrose were tested in different concentrations (2%, 4%, and 5%) and their effect on size, PDI and encapsulation efficiency was evaluated. The process of freeze drying was carried out at −45°C and 0.250 mBar pressure for 12 h. The same procedure was applied for freeze drying of control samples without cryoprotectant. Freeze dried sample was reconstituted in distilled water (2 ml) for further studies.

### 2.6 Stability Study

The stability studies of methotrexate loaded Eudragit S100 nano-suspension was evaluated through storing nano-suspension at different conditions, i.e., 25°C and 4°C for 6 months. The samples were placed in closed glass vials throughout the storage phase. Analysis of the nano-formulations was performed to assess PDI, size of particle and % EE of freshly prepared nano suspensions and samples kept at stability conditions. Samples were evaluated in triplicate for each storage condition after 1, 3, and 6 months of storage.

### 2.7 Statistical Analysis

Different statistical parameters like mean (X̄), standard deviation (SD) and relative standard deviation (% RSD) were used for quantifying methotrexate in mice. The data was evaluated by student’s t-test for assessing significance of difference (*p* ≤ 0.05) among means of treatments.

## 3 Results and Discussion

Objective of the research was to prepare pH sensitive nanoparticles formulations of methotrexate for oral targeted drug delivery. Eudragit^®^ S100 was used along with different emulsifier (POL, PVA, SDS, and CTAB in different concentrations as 0.125%, 0.25%, and 0.5%). Over seventy formulations were prepared and were thoroughly assessed for size of their particle size, shape, zeta potential, encapsulation efficiency, poly dispersibility index and *in vitro* drug release and freeze drying.

### 3.1 Optimization of Process Variables

Nanoparticles were prepared through modified emulsion/solvent evaporation technique. This method includes the following steps;

• Solubilization of polymer and drug in organic medium
• Addition of drug polymer mixture into surfactant solution (aqueous phase)
• Sonication through probe sonicator
• Evaporation of organic solvent resulting drug encapsulation by polymer

Surfactant has a significant role in formulation development by emulsion-solvent evaporation method. Different surfactants (POL, PVA, SDS, and CTAB) were evaluated and optimal results were obtained with POL and PVA. Different formulations of nanoparticles were prepared with these two surfactants, i.e., POL and PVA in different concentration (0.5%, 0.25%, and 0.125%). Different parameters were investigated in order to get optimum size and encapsulation efficiency of nanoparticles.

#### 3.1.1 Optimization of Sonication Speed

The effect of homogenization speed on characteristics of nanoparticle was studied by varying sonication amplitude and other parameters (drug concentration, polymer conc., and surfactant concentration) were kept constant. Nanoparticles prepared at lower sonication amplitude (60%) resulted in larger particle as compared with the higher sonication amplitude (99%) that results in smaller particles (Khatik et al., 2013) as shown in Table 3. High amplitude emulsification results in smaller emulsion globules leading to formation of nanoparticles of smaller size. More energy is released with increasing the homogenization amplitude, resulting in fast dispersion of organic phase, resulting in smaller sized nanoparticles (Madani et al., 2018). The encapsulation efficiency increased when emulsification speed was increased which might be due to less turbulent and unidirectional flow at lower sonication speed. Higher encapsulation efficiency at higher sonication speed can be attributed to greater surface area because of smaller size globules of the organic phase. At higher surface area drug polymer interface is larger, resulting in higher encapsulation efficiency and vice versa at lower amplitude.

**TABLE 3:** Effect of sonication speed and sonication time on particle size and encapsulation efficiency.

Parameters	Code	Sonication speed (%)^a^	Drug: polymer	Particle size (nm)	Zeta potential (mV)	PDI	% Encapsulation efficiency
Sonication speed	FMVs1	60	1:10	259 ± 1.15	−0.344 ± 0.10	0.185 ± 0.015	39.95 ± 0.7
	FMVs2	80	1:10	165.7 ± 1.85	−0.163 ± 0.11	0.215 ± 0.010	61.42 ± 2.1
	FMVs3	99	1:10	188.6 ± 3.1	−1.85 ± 0.15	0.233 ± 0.008	65.04 ± 0.8
Sonication time	FMVt4	1	1:10	270 ± 2	0.171 ± 0.14	0.207 ± 0.022	37.92 ± 2.7
	FMVt5	3	1:10	188.6 ± 3.1	−1.85 ± 0.15	0.233 ± 0.008	47.04 ± 1.9
	FMVt6	6	1:10	192.5 ± 2.83	−2.48 ± 0.20	0.237 ± 0.006	49.3 ± 1.5
Effect of surfactant	FMVr7	Poloxamer-407	1:10	165.7 ± 1.85	−0.163 ± 0.11	0.215 ± 0.010	61.42 ± 2.1
	FMVr8	SDS	1:10	1,108 ± 969.22	−53.1 ± 3.5	1 ± 0	2.55 ± 1.1
	FMVr9	CTAB	1:10	133.4 ± 8.51	51.4 ± 0.34	0.782 ± 0.07	33.19 ± 2.5
	FMVr10	PVA	1:10	187.7 ± 1.09	−0.59 ± 0.36	0.437 ± 0.020	59.01 ± 3.6

a Speed of the instrument can be measured in terms of percentage.

#### 3.1.2 Optimization of Sonication Time

Increase in sonication time increases energy input during emulsification (Khatik et al., 2013). The sonication time was varied from 1 to 6 min while other experimental conditions (drug concentration, polymer concentration, and surfactant concentration) were kept constant, to describe the effect of sonication time on size of nanoparticle. It was noticed that as with increasing sonication time (from 1 to 3 min) resulted in a decrease in the size of nanoparticles, as summarized in Table 3. Furthermore, with the increase in time of sonication from 3 to 6 min, the size of particle increases. It may be due to agglomeration or de-emulsification process.

#### 3.1.3 Optimization of Surfactant

Type and concentration of surfactant play an important role during emulsification and controls particle size through decreasing surface tension. Different types of surfactants having different HLB values were assessed for their possible effect on size of nanoparticle and process yield. Cetyl trimethylammonium bromide (CTAB) gave lowest particle size as compared with POL and sodium dodecyl sulfate (SDS). However the encapsulation efficiency was lower as compared with POL. Nanoparticles formulation was assessed by employing four different surfactants, that is, POL, PVA, SDS, and CTAB (Table 3). The innate characteristic of surfactants especially that adsorb at the interface reduces the surface tension and results in smaller size nanoparticles. This can be further described by the increased viscosity of surfactant solution which stabilizes the system and preventing coalescence of the particles (Nasef et al., 2017).

#### 3.1.4 Optimization of Drug to Polymer Ratio

The mean particle size of nanoparticles increased with increasing the quantity of drug (Table 4). It was observed that increased quantity of drug led to more viscous disperse phase, resulting in larger particle size (Madani et al., 2018). The encapsulation efficiency showed a slight downward tendency with increase in drug quantity in the formulation. The encapsulation efficiency of nanoparticles is influenced by drug miscibility in polymer and polymer-drug interactions.

**TABLE 4:** Effect Drug concentration on particle size and encapsulation efficiency.

Code	Drug (mg)	Eudragit S100 (mg)	Particle size (nm)	Zeta potential (mV)	PDI	% Encapsulation efficiency
FMVd11	2	30	174.6 ± 3.00	−5.64 ± 0.36	0.227 ± 0.017	70 ± 3.4
FMVd12	4	30	175.5 ± 0.43	1.58 ± 0.38	0.182 ± 0.02	30.19 ± 1.3
FMVd13	6	30	207.5 ± 13.09	1.11 ± 0.26	0.415 ± 0.06	56.96 ± 2.9

### 3.2 Characterization of Methotrexate Loaded Nanoparticles

#### 3.2.1 Particle Size

Three different concentrations of Eudragit S100 (10, 20, and 30 mg) were used, keeping the drug concentration constant (2 mg), the size of particle, encapsulation efficiency and zeta potential were evaluated, results are presented in Table 5. Formulations were prepared with Eudragit S100, through emulsion solvent evaporation method, using emulsifier POL, and PVA in different concentrations (0.5%, 0.25%, and 0.125%). As ratio of drug to polymer was raised from 1:5 to 1:15 (by weight), increase in particle size and the reason was increased in viscosity of the polymer solution, leading to decreased dispersion of polymer solution into the aqueous phase (Qindeel et al., 2019).

**TABLE 5:** Characterization of methotrexate nanoparticles using Eudragit S100.

Code	Drug:polymer	Particle size (nm)	Zeta potential (mV)	PDI	Amount encapsulated (mg/ml)	% Encapsulation efficiency
MSX1	1:5	114.6 ± 11.2	−2.92 ± 0.27	0.389 ± 0.22	0.78	39.26 ± 4.5
MSX2	1:5	109.7 ± 2.50	−2.36 ± 1.15	0.385 ± 0.006	0.73	36.81 ± 2.1
MSX3	1:5	135.1 ± 1.89	−1.59 ± 0.27	0.245 ± 0.016	0.65	32.68 ± 3.2
MSX4	1:10	171 ± 5.15	−0.10 ± 0.18	0.373 ± 0.045	1.14	63.02 ± 1.5
MSX5	1:10	165.7 ± 1.85	−0.163 ± 0.11	0.215 ± 0.010	1.228	61.42 ± 2.1
MSX6	1:10	185.9 ± 2.96	−3.54 ± 0.30	0.264 ± 0.014	1.01	50.7 ± 1.9
MSX7	1:15	231.9 ± 0.51	−5.06 ± 0.64	0.372 ± 0.018	1.31	65.89 ± 2.4
MSX8	1:15	174.6 ± 3.00	−5.64 ± 0.36	0.227 ± 0.017	1.4	70 ± 1.2
MSX9	1:15	202.6 ± 1.20	−2.28 ± 0.15	0.235 ± 0.008	1.38	69.19 ± 1.8
MSP1	1:5	776 ± 56	−0.23 ± 0.09	0.065 ± 0.08	1.40	70.18 ± 3.6
MSP2	1:5	492 ± 9.6	−11.5 ± 0.47	0.362 ± 0.04	1.20	60.01 ± 1.5
MSP3	1:5	1,190 ± 49	−0.161 ± 0.21	0.114 ± 0.13	0.98	49.36 ± 2.9
MSP4	1:10	981 ± 41.27	−1.97 ± 0.88	0.301 ± 0.45	1.62	81.45 ± 2.4
MSP5	1:10	872 ± 21.70	−0.841 ± 0.05	0.235 ± 0.08	1.39	69.51 ± 1.7
MSP6	1:10	1,336 ± 35.65	−1.05 ± 0.28	0.189 ± 0.46	1.08	54.47 ± 1.9
MSP7	1:15	1,241 ± 28.27	−0.67 ± 0.34	0.22 ± 0.11	1.82	91.31 ± 2.2
MSP8	1:15	1,118 ± 21.83	−0.355 ± 0.47	0.384 ± 0.35	1.51	75.82 ± 2.8
MSP9	1:15	1,528 ± 10.71	−0.796 ± 0.27	0.565 ± 0.33	1.20	60.08 ± 3.1

M, methotrexate; S, Eudragit S100; P, poly vinyl alcohol; X, Poloxamer-407.

**FIGURE 2:**
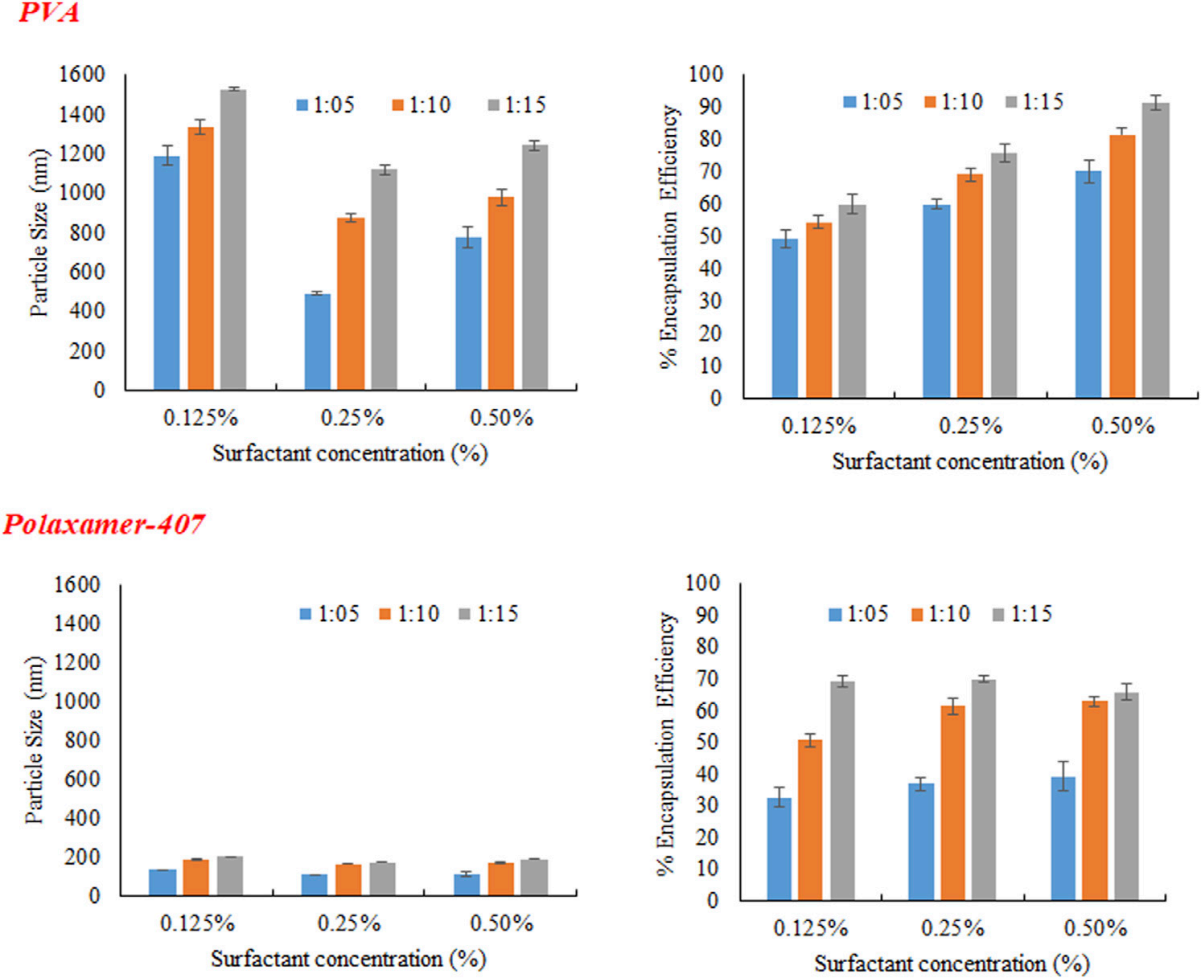
Effect of Eudragit S100 conc. on **(A)** particles size **(B)** % Encapsulation efficiency of MTX nanoparticles using different emulsifiers (PVA and Poloxamer-407).

**FIGURE 3:**
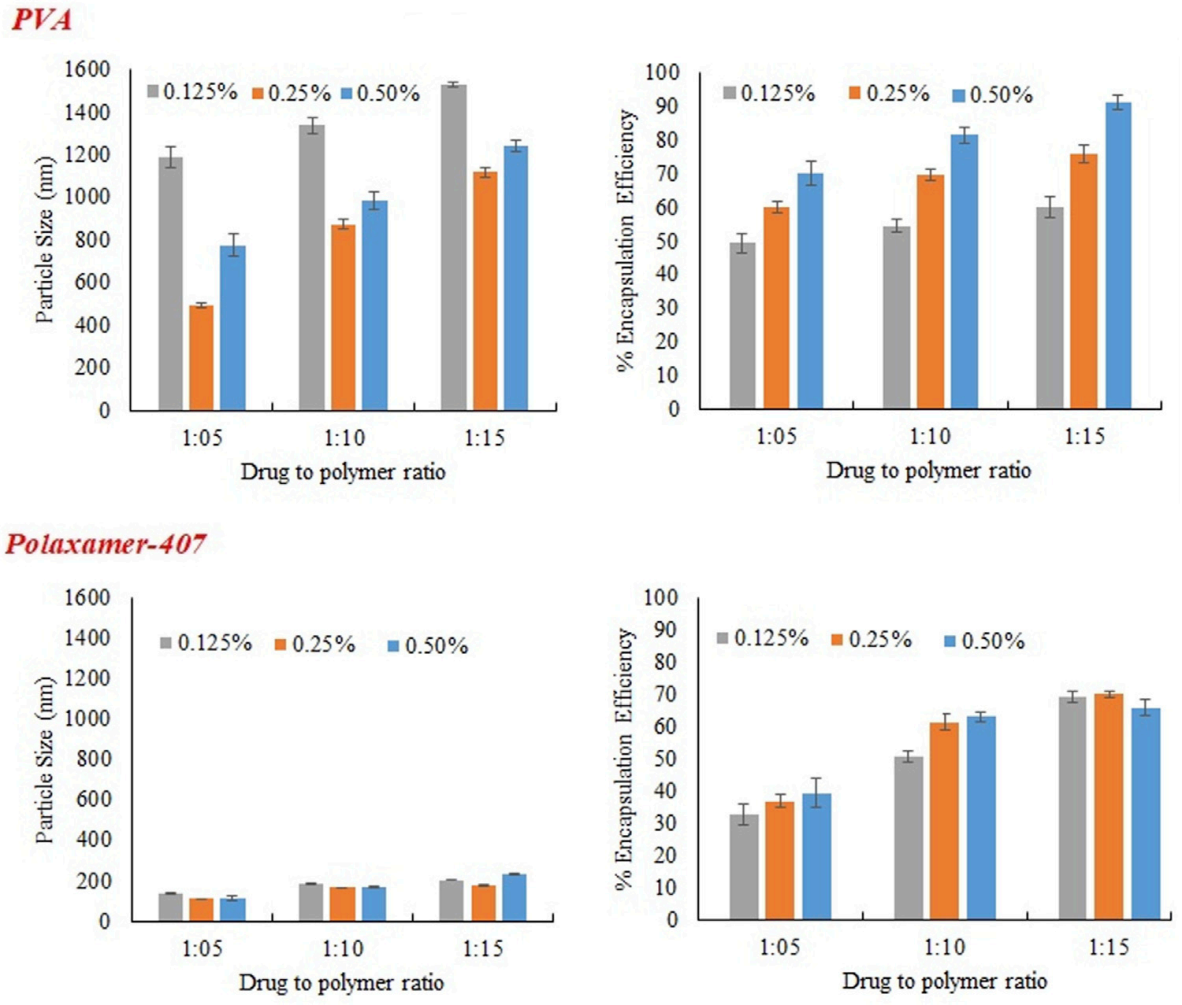
Effect of concentration of emulsifier (PVA and Poloxamer-407) on particles size, and encapsulation efficiency of MTX nanoparticles using Eudragit S100.

As evident from Figure 2 that irrespective of type and concentration of the surfactant increase in polymer concentration increases size of the nanoparticles. It was noticed that as concentration of POL was increased from 0.125% to 0.25%, the size of NPs was decreased as shown in Figure 3. This may be because of the reason that increase in surfactant concentration causes reduction in surface tension and facilitating particles partition. The reduction in the particle size is usually supplemented by a quick increase in the surface area (Madani et al., 2018). Further increasing the POL concentration to 0.5%; the size of particle increases which may be due to increased interaction between the molecules of stabilizers, leading to increased adsorption of surfactant on surface of nanoparticle forming multiple layer (Sharma et al., 2016).

PVA was used in three different concentration (0.125%, 0.25%, and 0.5%) as a stabilizer. At low concentration (0.125%) particles of larger size were obtained initially because of non-uniform and poor PVA coating onto freshly prepared emulsion droplets. Increasing the concentration of PVA from 0.125% to 0.25%, there was a sharp decrease in size of particle as obvious from Figure 3. The sharp reduction in size of particles may be due to decrease interfacial tension and prevention of droplet agglomeration. Increasing the concentration of PVA further up to 0.5% leads to increase in particle size. The size of particle decreases as concentration of surfactant increases in aqueous phase up to certain limit may be because of the lining up of surfactant molecule at the interface leading to a decrease in interfacial tension. Further increasing the concentration of surfactant in external phase above certain limits causes an increase in size of particle and this might be because of the increased viscosity and a reduction in net shear stress resulting in the larger particles formation.

#### 3.2.2 Zeta Potential

Zeta potential was evaluated through electrophoretic mobility of the particles. It is a key parameter to assess the *in-vivo* properties as well stability of nanoparticles. Generally negative zeta potential was obtained for MTX with Eudragit S100 because of the free acrylic acid groups available on Eudragit S100 (Jang et al., 2019). The zeta potential varied between −0.10 ± 0.018 and −11.50 ± 0.47 for MTX nanoparticles but there was no specific decrease or increase pattern with increasing or decreasing Eudragit S100 as shown in Table 5. There was no specific pattern for decrease or increase is followed by enhancing poloxamer 407 concentration.

#### 3.2.3 Encapsulation Efficiency

The concentration of polymer in organic medium effects encapsulation efficiency, significantly. The % EE improved by increasing the concentration of polymer from 1:05 to 1:15 with respect to drug. Highest encapsulation efficiency was obtained when ratio of drug to polymer was 1:15. The viscosity of organic phase increased with higher concentration of polymer, resulting in more resistance to drug diffusion from organic to aqueous phase, which led to higher encapsulation efficiency (Madani et al., 2018). At higher polymer concentration, polymer precipitation time decreases, which reduces drug diffusion out of nanoparticles. Concentration of PVA has directly proportional effect on % EE as shown in the Figure 3. It may be due to augmented viscosity of external medium as a result of thick layer of stabilizer and minimum diffusion of drug to external aqueous medium. Similarly, higher encapsulation efficiency was obtained with increasing the concentration of surfactant (POL) which might be due to stronger binding contacts between drug and polymer.

#### 3.2.4 X-Ray Diffraction

**FIGURE 4:**
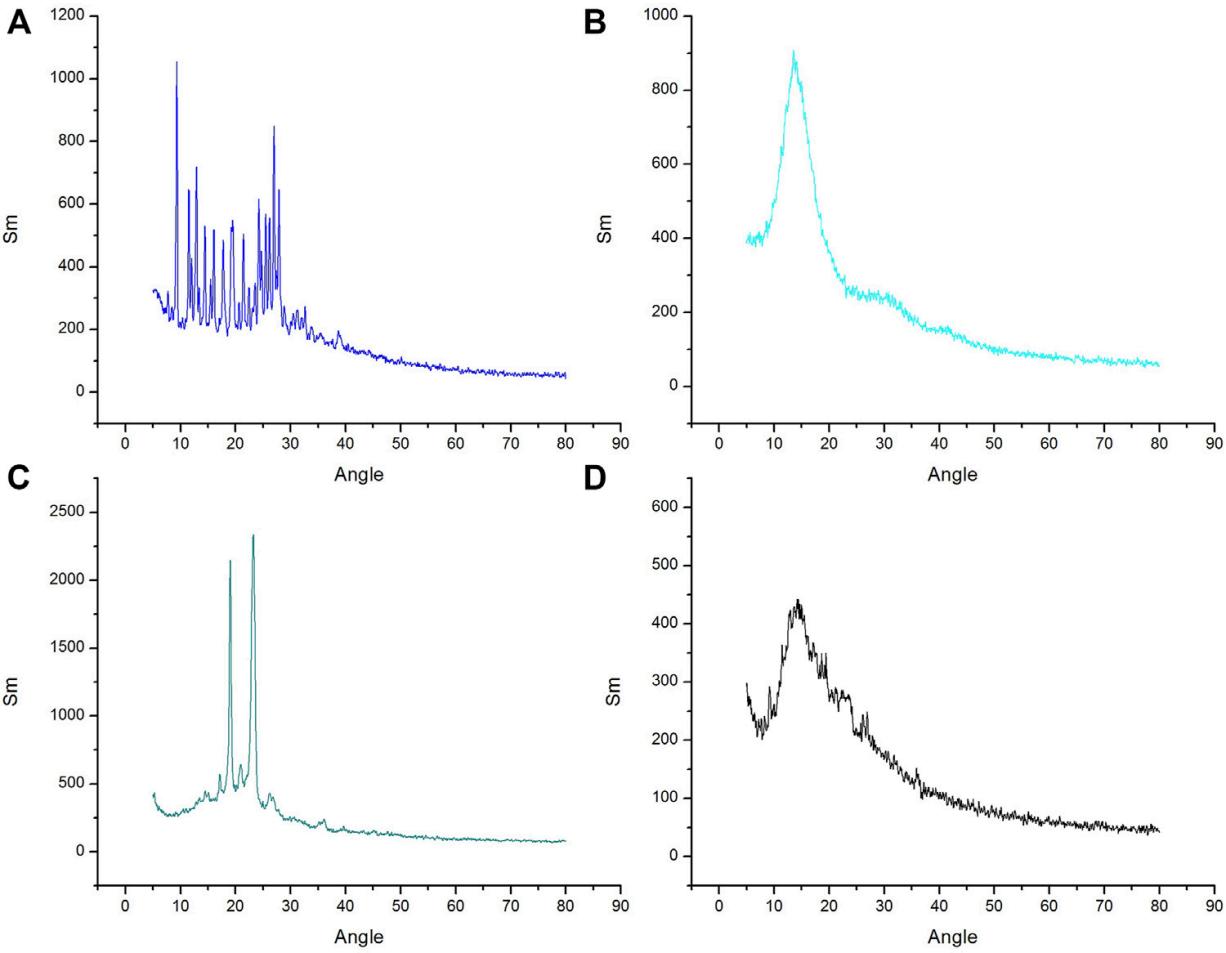
XRD curve of **(A)** MTX, **(B)** Eudragit S100 **(C)** Poloxamer-407 and **(D)** MTX- Eudragit S100 nanoparticles (MSX8).

X-ray diffraction was applied to study the crystalline nature of drug. The X-Ray diffractometer, was utilized to study the XRD patterns of excipients, drugs and drug-loaded nanoparticles. All the XRD patterns were recorded at ambient temperature at diffraction angle of 2θ in a range of 3^°^–80^°^. The diffractogram of pure MTX comprised of numerous characteristic sharp peaks. The most promising peaks of MTX were present at 7.90, 10.6, 11.8, 13.1, 14.5, 19.3, 21.6, and 28, confirming crystalline nature of pure MTX. The XRD characteristic peak for POL was determined at 18° and 23.2° (2 Theta) and there was no peak for Eudragit S100 as shown in Figure 4. In case of MTX-Eudragit S100 nanoparticles with POL no peaks were observed indicating that the drug is in amorphous state.

### 3.3 Selection of Optimal Formulations

**FIGURE 5:**
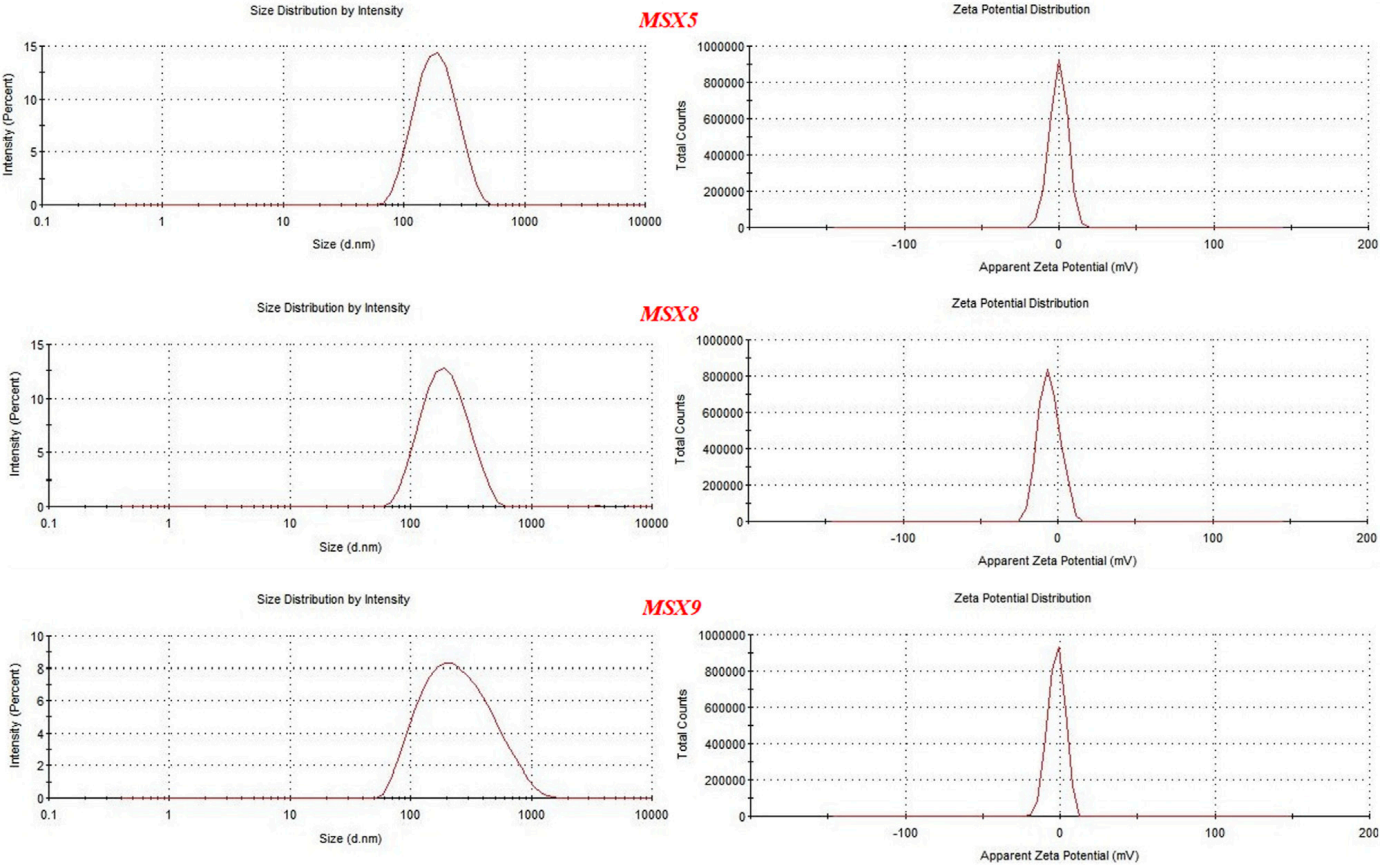
Graphs showing particle size and zeta potential of optimal formulation of methotrexate loaded nanopatricles.

Different formulations of nanoparticles were developed using Eudragit S100 as polymer and POL and polyvinyl alcohol. For the selection of optimized formulations three main parameters were considered, i.e., particle size, encapsulation efficiency, and zeta potential and on this basis three formulations were selected, including MSX5, MSX8, and MSX9 (In nomenclature of the formulations, M denotes methotrexate, S denotes Eudragit S100 and X denotes Poloxamer-407), as shown in Table 6. Size distribution and zeta potential curves of the optimal formulations are presented in Figure 5.

**TABLE 6:** Characteristics of optimized formulations of methotrexate.

Code	Particle size (nm)	Zeta potential (mV)	PDI	Amount encapsulated (mg/ml)	% Encapsulation efficiency
MSX5	165.7 ± 1.85	−0.163 ± 0.11	0.215 ± 0.010	1.228	61.42 ± 2.1
MSX8	174.6 ± 3.00	−5.64 ± 0.36	0.227 ± 0.017	1.4	70 ± 1.2
MSX9	202.6 ± 1.20	−2.28 ± 0.15	0.235 ± 0.008	1.42	71.19 ± 1.8

**FIGURE 6:**
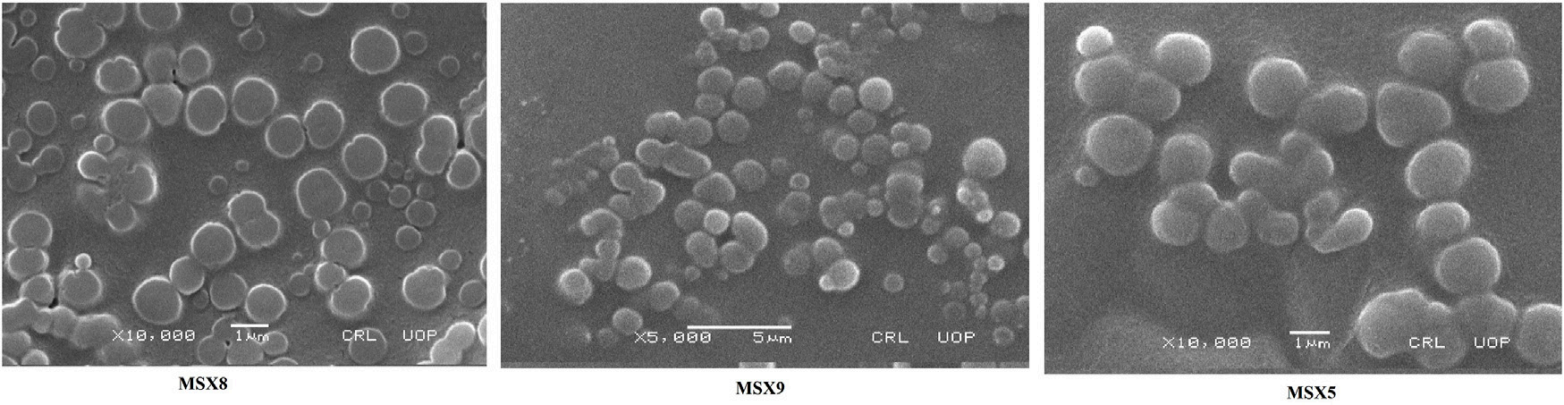
SEM images of methotrexate loaded nanoparticles prepared using Eudragit S100.

The morphology of nanoparticles is important in biodistribution, targeting drug to various organs and circulation time. Results of SEM images of the optimized formulation showed that nanoparticles obtained were spherical with smooth surfaces (Figure 6).

### 3.4 Freeze Drying of the Developed Nanoparticles

The smaller particle size, increase surface area and colloidal nature of nanoparticles may lead to the physical instability, including aggregation and fusion of particle. Chemical instability includes hydrolysis of polymer and drug leakage from nanoparticles. So as to minimize problem of instability freeze drying or lyophilization process is used to remove water from the developed formulation and get dry nanoparticles. In this study, the optimized formulations were freeze dried with or without the addition cryoprotectants. Two different cryoprotectants; mannitol and sucrose were evaluated in three different concentrations, i.e., 2%, 4%, and 8%. The samples which were freeze dried were reconstituted with purified water (2 ml). The impact of the cryoprotectants on the various properties of the nanoparticles is shown in Table 7. The mannitol (2%) showed very slight variations in size of particle and PDI and hence selected as a cryoprotectant.

**TABLE 7:** The impact of Freeze drying on methotrexate nano-formulations with different cryoprotectant (Mannitol and sucrose).

Status	Parameter	MSX5	MSX8	MSX9
Initial	Size (nm)	165.7 ± 1.85	174.6 ± 3.00	202.6 ± 1.20
	PDI	0.215 ± 0.010	0.227 ± 0.017	0.235 ± 0.008
Without cryoprotectant	Size (nm)	218.5 ± 16.74	242.7 ± 9.78	258.5 ± 13.85
	PDI	0.271 ± 0.024	0.291 ± 0.013	0.243 ± 0.004
2% Sucrose	Size (nm)	249.5 ± 10.7	265.3 ± 15.31	293.4 ± 16.25
	PDI	0.251 ± 0.02	0.316 ± 0.05	0.28 ± 0.02
4% Sucrose	Size (nm)	258.6 ± 18.04	289.5 ± 16.08	306.8 ± 14.38
	PDI	0.423 ± 0.02	0.321 ± 0.04	0.338 ± 0.02
8% Sucrose	Size (nm)	318.3 ± 12.73	322.1 ± 15.69	340.9 ± 19.95
	PDI	0.301 ± 0.02	0.268 ± 0.05	0.297 ± 0.04
2% Mannitol	Size (nm)	184.2 ± 10.30	188.9 ± 9.37	209.3 ± 11.64
	PDI	0.248 ± 0.03	0.173 ± 0.04	0.341 ± 0.01
4% Mannitol	Size (nm)	199.2 ± 9.17	200.3 ± 14.81	226.1 ± 13.70
	PDI	0.339 ± 0.04	0.159 ± 0.07	0.303 ± 0.02
8% Mannitol	Size (nm)	231.1 ± 15.74	239.3 ± 11.95	269.4 ± 18.95
	PDI	0.225 ± 0.05	0.283 ± 0.05	0.199 ± 0.07

### 3.5 Stability Study

For evaluation of stability, the developed optimal formulation was stored at 4°C and 25°C and their encapsulation efficiency, particle size and PDI were evaluated. The results are shown in Table 8 indicating slight differences on size, PDI and EE of nanoparticle when stored at 4°C for 6 months. The kinetic energy at low temperature is reduced and therefore particles contact is prevented leading to decreased particles aggregation (Chacon et al., 1999). When the formulations were stored at 25°C, prominent changes were observed in PDI, % EE and particle size. Thus the formulations may be kept at 4°C to avoid variation in particle size, PDI and % EE.

**TABLE 8:** Results of stability studies of methotrexate nanoparticles.

Time	Code	Stored at 4°C			Stored at 25°C		
		Size (nm)	PDI	% EE	Size (nm)	PDI	% EE
Day 01	MSX5	165.7	0.215	61.42	167.6	0.202	61.42
	MSX8	174.6	0.227	70	175.1	0.223	70
	MSX9	202.6	0.235	71.19	203.5	0.252	71.19
1 Week	MSX5	167.7	0.218	61.42	169.6	0.252	61.42
	MSX8	175.2	0.237	70	179.1	0.224	70
	MSX9	204.4	0.222	71.19	204.5	0.301	71.19
1 Month	MSX5	166.4	0.236	60.52	171.9	0.302	58.35
	MSX8	177.2	0.247	69.5	180.3	0.281	68.51
	MSX9	204.6	0.322	70.01	207.7	0.311	68.19
3 Month	MSX5	169.9	0.253	59.65	176.8	0.322	56.43
	MSX8	176.6	0.221	68.91	185.5	0.301	67.01
	MSX9	206.4	0.266	70.01	213.3	0.334	67.29
6 Month	MSX5	170.8	0.299	58.89	175.2	0.311	57.35
	MSX8	179.5	0.244	68.02	187.6	0.312	67.01
	MSX9	207.1	0.282	69.54	214.5	0.339	67.17

### 3.6 *In Vitro* Drug Release

**FIGURE 7:**
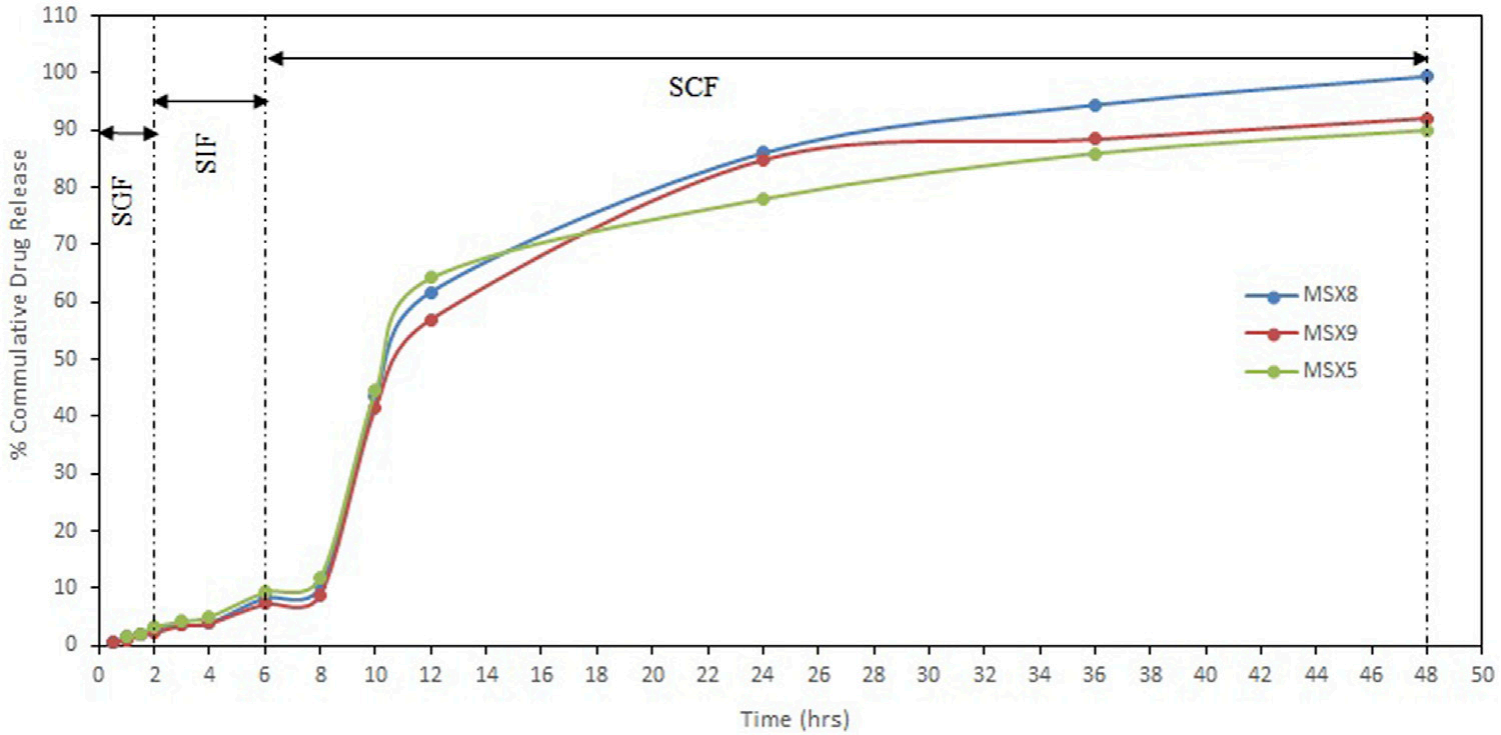
*In-vitro* release profile of methotrexate from eudragit S100 nanoparticles in different simulated GI tract fluids (SGF, simulated gastric fluid; SIF, simulated intestinal fluid; SCF, simulated colonic fluid).

*In vitro* release of MTX from polymeric nanoparticles was studied by dialysis bag diffusion method in a shaking water bath. Dissolution media consisted of simulated gastric fluid (SGF) (0.1 N HCl pH 1.2), simulated intestinal fluid (SIF) (phosphate buffer pH 6.5), and simulated colonic fluid (SCF) (phosphate buffer pH 7.4). The results of “*in-vitro*” release are presented in Figure 7.

The pH-dependent drug release from NPs was evaluated between the pH of the medium in the range of 1.2, 6.5, and 7.4, which resemble the stomach, small intestine, and colon pH, respectively. Release of methotrexate during the first 2 h in SGF (resembling stomach pH 1.2) from Eudragit S100 nanoparticles was 2.22%–3.26%. The release of drug in simulated intestinal fluid (resembling intestinal pH 6.5) for further 4 h is about 7.27%–9.34% from Eudragit S100 nanoparticles. The release of methotrexate from Eudragit S100 nanoparticles during the next 42 h in simulated colonic fluid (resembling colonic pH 7.4) is about 90.01%–99.41%. The pH dependent release profile of Eudragit S100 shows a slow drug release at acidic pH that ascended to a quick release upon changing the pH. The Eudragit S100 prevents the drug release at gastric pH and drug is released as in neutral and alkaline medium which may be due to the carboxyl group of Eudragit that ionize in neutral to alkaline media (Matlhola et al., 2015).

## 4 Conclusion

The present study was carried out to develop pH sensitive, targeted polymeric nanoparticles of methotrexate using Eudragit S100 in combination with different surfactants by modified emulsion solvent evaporation technique. The prepared nanoparticles showed good physicochemical properties in terms of size, zeta potential, PDI, and encapsulation efficiency. The prepared nanoparticles were suitable for colon drug targeting. Stability studies showed that optimized nanoparticles were quite stable when stored at 4°C for 6 months. The *in-vitro* release study showed that initially the drug released through diffusion and followed by the erosion of polymeric chains. It is concluded that the desired properties of nanoparticles (particle size and surface charge) can be achieved by utilizing proper type and concentration of surfactant. Furthermore, emulsion solvent evaporation technique was proven to be highly effective for preparation of nanoparticles of hydrophobic drugs.

## Data Availability

The original contributions presented in the study are included in the article/supplementary material, further inquiries can be directed to the corresponding authors.
